# Soiling Effect Mitigation Obtained by Applying Transparent Thin-Films on Solar Panels: Comparison of Different Types of Coatings

**DOI:** 10.3390/ma14040964

**Published:** 2021-02-18

**Authors:** Małgorzata Rudnicka, Ewa Klugmann-Radziemska

**Affiliations:** Department of Energy Conversion and Storage, Faulty of Chemistry, Gdansk University of Technology, 80-233 Gdańsk, Poland; malgorzata.rudnicka@pg.edu.pl

**Keywords:** photovoltaics, dust accumulation, hydrophobic, hydrophilic, anti-dust

## Abstract

Dust accumulation on the front cover of solar panels is closely linked to location and orientation of photovoltaic (PV) installation. Its build-up depends on the module tilt angle, frequency of precipitation, humidity, wind strength and velocity, as well as grain size. Additionally, soil composition is determined by solar farm surroundings such as local factories, agricultural crops, and traffic. Over time, molecules of atmospheric dust agglomerate on top of each other and cause gradual reduction in generated energy. Manual cleaning techniques are required to restore working conditions of PV installation to their original conditions; however, they are time consuming and may lead to damage of the glass coverage. Therefore, implementing a different approach by utilizing self-cleaning and anti-dust coatings on front covers of module surfaces is thought of as a competitive manner of cleansing. Based on the varying properties of such thin-films, a division was made into hydrophobic, hydrophilic, and anti-dust coatings. In this article, the authors would like to present a comprehensive review of those types of transparent films. Moreover, a few hydrophobic coatings available on the Polish market were analyzed by applying them on glass tiles and covering them with three types of dust.

## 1. Introduction

Solar energy is the steady growing green energy source with both large companies as well as individual people willing to invest in this market. As the need to reduce carbon footprints is common in our everyday lives, one should not be surprised that photovoltaic establishment tries to mitigate any drawbacks connected to solar farms. One such difficulty is connected to pollutant deposition on photovoltaic (PV) module surfaces, which directly corresponds to the lowered energy yield possible to obtain from installation. Soil build-up progressing over time causes the reduction of the glass transparency of the front module cover [1,2,3]. This, in turn, leads to decreases of solar irradiance reaching semiconductor cells and decrements in current output [4,5,6,7]. Such effects could be partially mitigated if precipitation occurs and washes off a part of the accumulated pollution [8].

The rate of dust accumulation on top of the front glass coverage of PV modules is closely linked to the tilt angle, latitude, and environmental surroundings in the close proximity of solar power sources [9]. The more horizontal the tilt angle, the higher the speed of soil settlement. Additionally, dust molecules tend to gather more at the bottom of PV modules because of gravitational forces dragging them down [10]. Any occurrence of heavy wind and storm or precipitation would likely cause a regain in efficiency [11,12]. On the other hand, frequent sand storms in desert regions negatively impact the power generation of solar energy, as they lead to complete coverage of glass surfaces, with sand almost completely cutting off sun radiation reaching solar cells [13]. Similarly, a sparse amount of rain or high levels of humidity cause a formation of a cement-like layer, which dries into to a hard shell on the glass surface [9].

Dust properties, namely, the size, shape, mass, and composition, depend on the location of solar power plants. They can also be influenced by nearby industry, heavy traffic or agricultural crops [14]. The relations found between soiling tempo and particle size is that for grains with a smaller diameter, the loss of glass transmission is greater as such molecules can be stacked closely and block irradiance more efficiently [10].

Manufacturers handling worldwide sales outline in documents concerning conservation of PV modules that it is mandatory to clean them manually [15,16,17,18,19]. It may help if the installation is small; however, this can hardly be an optimal solution for solar plants consisting of dozens or more devices. Not only is it time-consuming but wasting of water ought to be avoided if possible, given much more frequent and prolonged periods of drought.

Addressing this issue, alternative cleaning methods are developed, namely, electrostatic, mechanical, and surface enhancement [20]. Electrostatic cleaning utilizes standing and traveling waves to expel dust from electrostatic fields. Standing waves move the dust downwards or upwards, and horizontal movement is achieved by traveling waves. Mechanical cleaning offers four methods—robotic, air-blowing, water-blowing, and ultrasonic. Blowing air and water also result in reducing the temperature of PV surfaces. Lastly, surface enhancement revolves around introducing an additional layer on top of the front glass coverage. It utilizes nanotechnology in order to design hydrophobic and hydrophilic thin films. Hydrophobic coatings allow water drops to roll down and carry away dirt residue. Hydrophilic ones reduce the amount of dirt via photo-catalytic reactions. This article aims at familiarizing the reader with the overview of the current state of knowledge in the field of thin films developed specifically for usage in the photovoltaic industry. Additionally, experimental analysis of a few hydrophobic coatings available on the Polish market was performed.

## 2. Hydrophilic Coatings

Hydrophilic coatings are characterized by their high surface energy. After spraying them with water they distribute it evenly over the whole surface and thus clean it. They are oftentimes based on silica particles [21]; however, the photo-catalytic effect may also be incorporated by utilizing titanium or wolfram [21,22].

**Figure 1:**
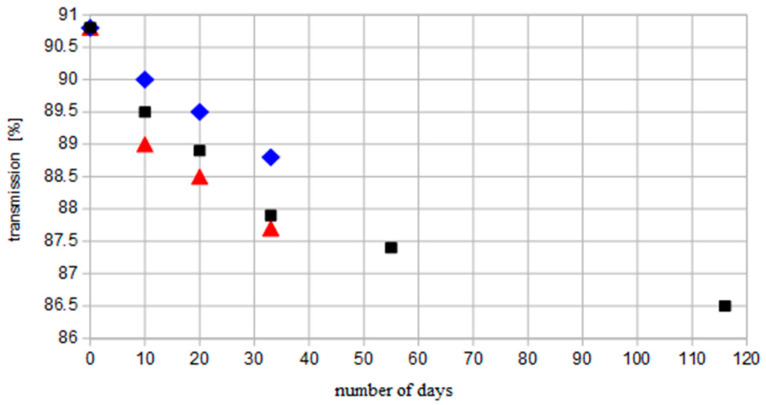
Graph of the average glass plate transmission as a function of the number of days (■) and transmission of the top (♦) and bottom (▼) part of the glass plate (based on: [23]).

Hee et al. analyzed the effect of dust settlement on glass slides without any coverage and discovered their transmission was reduced by 3% over the course of one month despite heavy rainfall occurring in Singapore [23]. The experiment was carried out for four months and after an initial steep linear decrease in glass transparency, this phenomena progressed more slowly for a day number over 32 (Figure 1). They added another conclusion such that the rate of transmission reduction was faster at the bottom of the glass tile, meaning the debris after precipitation tended to gather there due to gravitational force.

**Figure 2:**
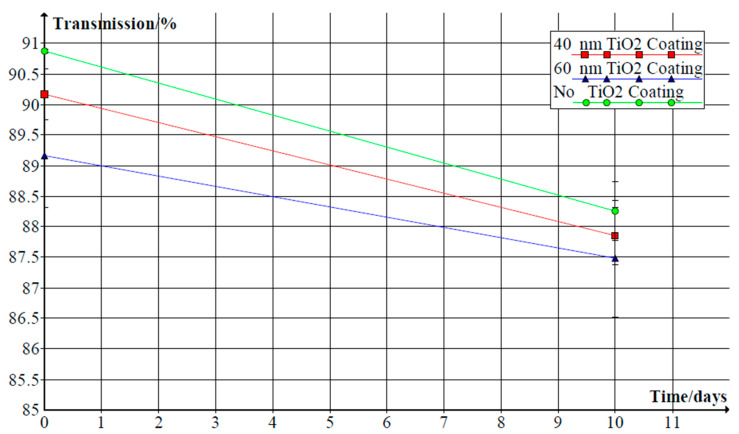
Transmittance spectrum for uncoated glass surface, pure TiO_2_ films, and TiO_2_/SiO_2_ composite films after placing them in a furnace at varying temperatures [23].

Later on, glass tiles were covered by a layer of TiO_2_ intended as a self-cleaning coating. Two different thickness levels were implemented—40 nm and 60 nm. Although a slower reduction of transmittance was observed for glass tile with 60 nm coating, it also had much lower initial transparency (Figure 2). The mitigation of dust settlement was hardly compensated in this situation, since the TiO_2_ layer negatively impacted glass transmittance.

**Figure 3:**
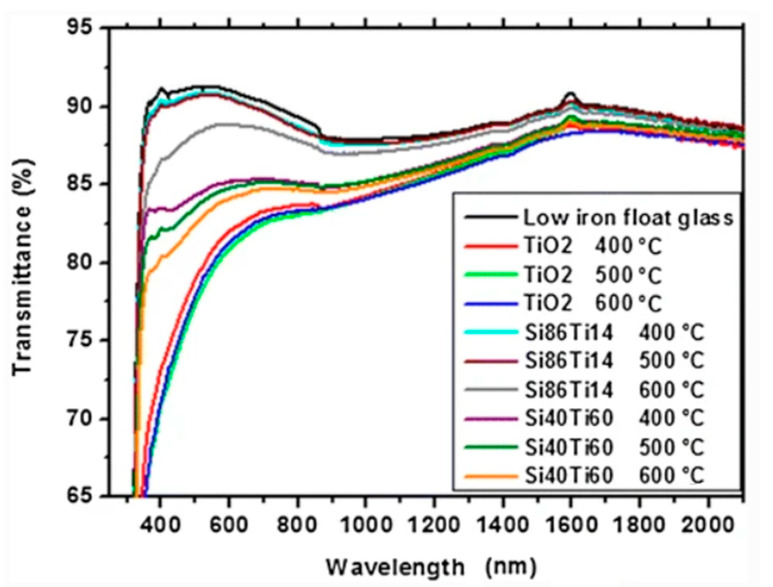
Transmittance spectrum for uncoated glass surface, pure TiO_2_ films, and TiO_2_/SiO_2_ composite films after placing them in furnaces at varying temperatures [24].

Introducing composites of TiO_2_/SiO_2_ was therefore proposed. Varying molar rates were tested by de Jesus, and the conclusion was drawn that adding molecules of silicon improved the transmittance of glass [24]. However, as is visible from Figure 3, the lesser the amount of titanium in the complex, the closer the resemblance of the transmittance function between the bare glass plate and the covered one.

**Figure 4:**
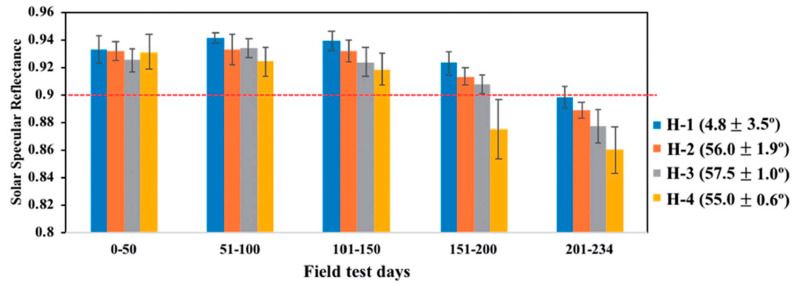
Solar specular reflectance as a function of time for mirrors with hydrophilic coatings with varying water contact angles (4.8°, 55°, 56°, 57.5°) [25].

Another utilization of hydrophilic properties included coatings prepared by Jang [25]. They were made out of SiO_2_ nanoparticle layers and a silica binder film between the nanoparticles and protected mirror surface. They showed complete water spreading, with a water contact angle (WCA) between 57.5° and 4.8°, and no negative impact on glass transparency was observed. Afterwards, they were subjected to external conditions for 234 days. The mirror with the lowest WCA layer experienced the least reduction in reflectance overall, but the difference between values for all mirrors was clearly visible after some exposure time, over at least 100 days (Figure 4).

Nabemoto proposed a photo-catalytic anti-soiling and hydrophilic layer that was obtained by modifying WO_3_ with partially hydrolyzed tetra-ethyl orthosilicate [26]. Its effect was observed on a surface of polymethylmethacrylate (PMMA), since such material is used as Fresnel lenses for concentrator photovoltaics. The difference was significant, as the reduction in the measured current output in the concentrator photovoltaic system was 9.6% without additional coating and 3.3% with coating.

The same approach was analyzed by another group from Japan. However, Sueto decided to further expand the number of added layers [27], and so PMMA was first spin-coated with an acrylic urethane capping layer, then with a nanograded intermediate layer, and finally with enhanced WO_3_ as a photocatalytic layer. It helped to shield PMMA from photocatalytic properties and to reduce reflectivity. The measured mass of dust agglomerated on the surface without coating exceeded 0.01 g, whereas for PMMA with the hydrophilic layer, it oscillated around 0.005 g.

**Figure 5:**
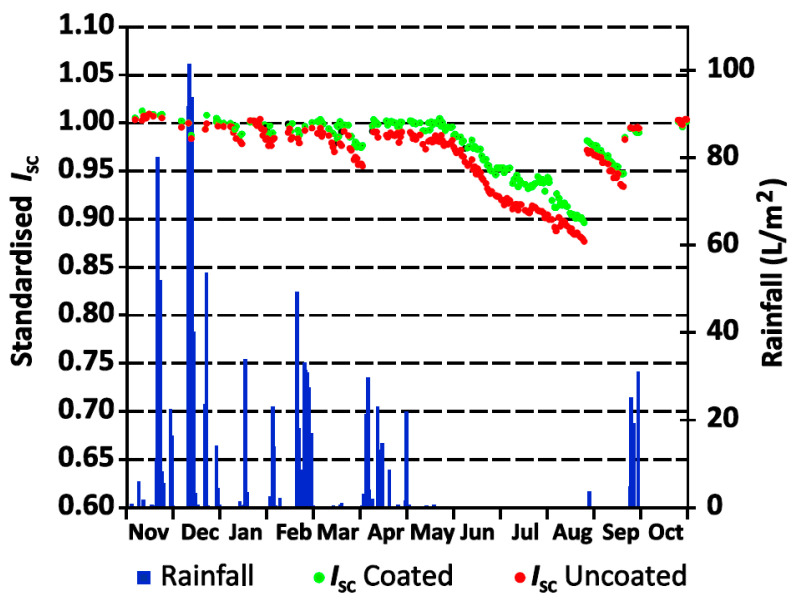
Short-circuit current values over the course of 12 months with daily rainfall data [28].

Testing performed on the PV modules was done by Canete [28]. A compound made of hybrid polymer and metal-oxide nanoparticles was flow-coated on the surface. Interestingly, initial power output was 1–1.5% higher after depositing this coating, suggesting better anti-reflectivity. Current–voltage characteristics were measured through one year, and modules were constantly kept outside; thus, they were subjected to real-time atmospheric conditions. Current values are presented in Figure 5 as a function of time, with additional insight into rainfall statistics. In the dry months of summer, the greatest difference between modules with and without coating was observed, corresponding to approximately 5%. 

## 3. Hydrophobic Coatings

Hydrophobic coatings exhibit a high water contact angle, over 90°, as well as low surface energy. As a result, they encourage dew and rain to form into spheres, causing such droplets to slide off of the surface and carry off any pollutant residue [29,30,31].

The approach utilizing SiO_2_ matrix with two sized particles (8 nm, 60 nm) was proposed by Bahattab [32]. After applying it onto a glass substrate, it was stated that transparency increased by a few percentile points. Annual exposure to the outside conditions in Saudi Arabia caused a 13% transmittance reduction for glass tiles with hydrophobic coatings and 19% for the surface without any film. The blowing of compressed air proved to be enough to almost completely clean the coated surface, since the transparency loss was 2%. However, it was not as successful for the other glass, as transparency loss reached 8%. Highly transparent silica nanoparticles synthesized with the addition of tetraethyl orthosilicate and ethanol were the aims of study carried out by Polizos [33]. They were deposited on top of the glass surface and left under external conditions for three months. Afterwards, it was stated that the transmittance of the uncoated tile was around 20–25% lower.

**Figure 6:**
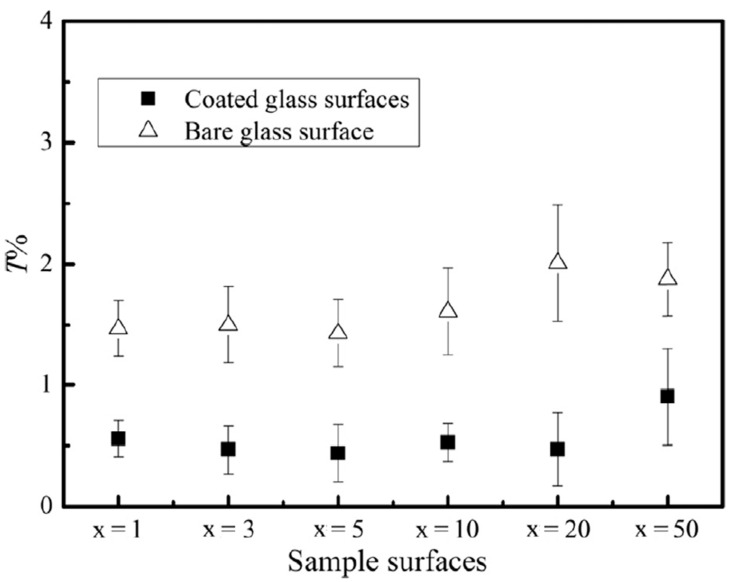
Transmittance loss for bare glass surface (Δ) and glass with (SS-SiO_2_)_x_ coating (■) [34].

Transparent hydrophobic coatings based on silica sol (SS) and SiO_2_ nanoparticles were proposed by Quan [34]. Their hydrophobic character could be adjusted by changing the x number of (SS-SiO_2_)_x_. Increasing the number x also resulted in heightened transmittance, and it was explained by the formation of nanovoids limiting reflection: x = 1 or x = 3 thin layers exhibit similar transparency as bare glass. For x > 5, transmittance rises a few percentile points and the sliding angle plummets. Bare glass and glass enhanced with thin layers were inspected after artificially blowing dust over each surface. From Figure 6, the conclusion can be drawn that the magnitude of the hydrophobic effect does not greatly impact the reduction in transmittance after polluting surfaces. Bare glass faces more than 1.5% transparency reduction and for glass with coatings, this number is less than 0.6%. Low surface energy and the course structure of such films is enough to hinder particle adhesion.

**Figure 7:**
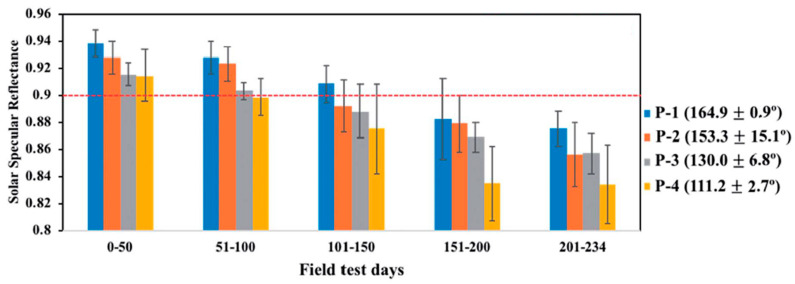
Solar specular reflectance as a function of time for mirrors with hydrophobic coatings with varying water contact angles (164.9°, 153.3°, 130°, 111.2°) [25].

The SiO_2_ nanoparticle layer and silica binder film between nanoparticles analyzed by Jang could also be altered to exhibit hydrophobic properties [25]. Additional thermal vapor deposition of fluorosilane over this thin film performed at a temperature of 120 °C set the water contact angle value between 111° and 165°. Afterwards, the testing surfaces were placed outside for 234 days, where mirror with the highest WCA layer outperformed other coatings (Figure 7).

**Figure 8:**
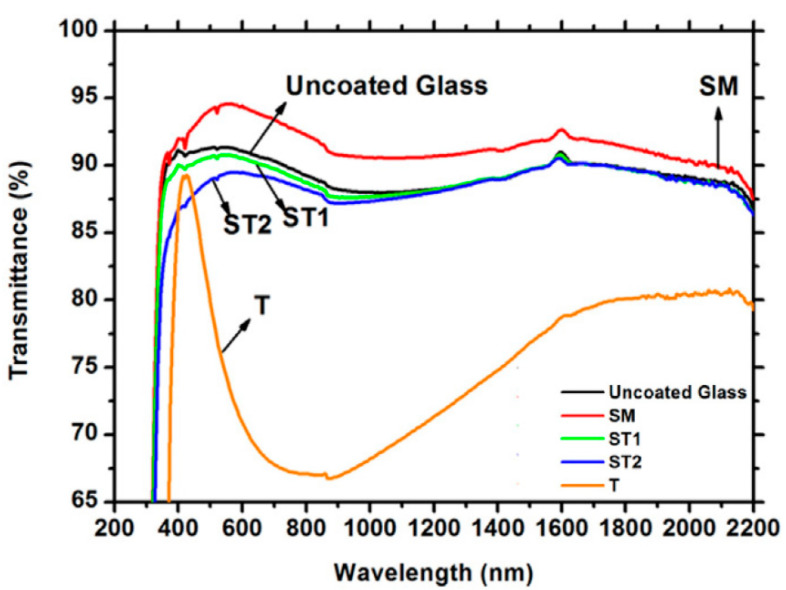
Transmittance spectrum for uncoated glass plate and glass with the layer of hydrophobic film (SM), hydrophilic Si_86_Ti_14_ (ST1), hydrophilic Si_40_Ti_60_ (ST2), and pure TiO_2_ (T) [35].

In another studies of de Jesus, SiO_2_ sol-gel treated with hexamethyldisilazane (referred to as SM) resulted in a hydrophobic thin film [35]. In comparison with their previous TiO_2_/SiO_2_ composite, it had much higher transparency, which is even superior to the bare glass (Figure 8). A soiling test was performed, but it was concluded that all glass plates with additional layers performed similarly with regards to dust adhesion and were easily cleaned with water.

## 4. A Study of Hydrophobic Coatings Available on the Polish Market

An additional experiment concerning hydrophobic coatings provided by certified Polish companies has been carried out on nine identical glass plates sized 0.13 m × 0.18 m. Each plate was cleaned with isopropyl alcohol, and then 2 mm of the liquid substance was sprayed directly on the surface and evenly distributed with a clean cloth. One glass was kept clean and without any coating for the comparative analysis. Table 1 contains names of applied products with corresponding glass plate numbers. Price conversion from PLN to USD was calculated via the pl.investing.com website on January 24th.

**Figure 9:**
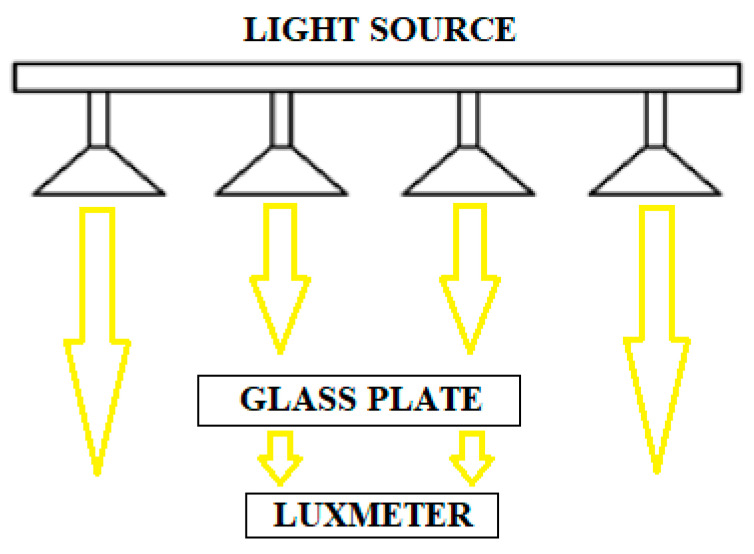
Illuminance measurement layout carried out by an Abatronic luxmeter.

Lighting intensity was measured with the aid of an luxmeter with catalog number AB-8809A manufactured by Abatronic company, that was placed behind the glass plate in accordance with Figure 9.

Firstly, illuminance values for clean glass plates were gathered. Further consecutive tests involved obtaining the lighting intensity for different pollutant types and layers of dust. Three various soils, later referred to as 1, 2, and 3, with four dust densities—4.3, 8.5, 12.8, and 21.4 g/m^2^—were deposited on each glass plate to calculate normalized illuminance for clean glass plates (1) and normalized illuminance after soil adhesion (2).

Every soil type applied in artificial dust experiments has been listed in Table 2. Soil 1 was taken directly from roads in the vicinity of Chemistry building, on Gdansk University of Technology (GUT) campus. The size of its grains differs, as some are much smaller with a diameter of 0.2 mm, whereas others reach around 0.45 mm. Soils 2 and 3 were gathered from sand beach areas in the close proximity of a walking pier and tram loop, both located in Gdańsk Brzezno district. They are much more uniform concerning grain size; for soil 2, the average diameter falls in the range of 0.23–0.36 mm and for soil 3, it is 0.22–0.33 mm.

## 5. Results and Discussion

**Figure 10:**
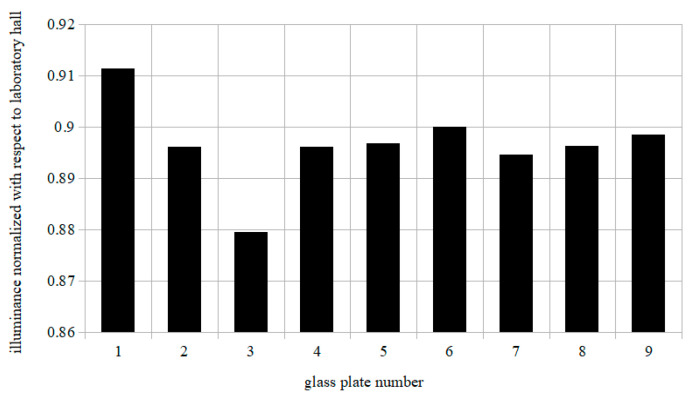
Illuminance measurement layout carried out by an Abatronic AB-8809A luxmeter.

Figure 10 presents the normalized illuminance for clean glass plates with respect to light intensity in the laboratory hall that oscillated around 330 lux at the beginning of the experiment. It should be noted that all of the glass plates caused an approximate 10% light intensity decrease, regardless of whether they were covered with coating, as this is attributed to transmittance reduction caused by the glass itself. Glass covered with Eco hydrophobic impregnate exhibited slightly lower illuminance loss than other slides with coatings; however, differences between the light intensity calculated between each plate were minimal and did not exceed 1% with regards to glass tile 6. Therefore, it may be stated that hydrophobic surface enhancement leads to a slight decrease of transparency and could be applied to photovoltaic modules.

**Figure 11:**
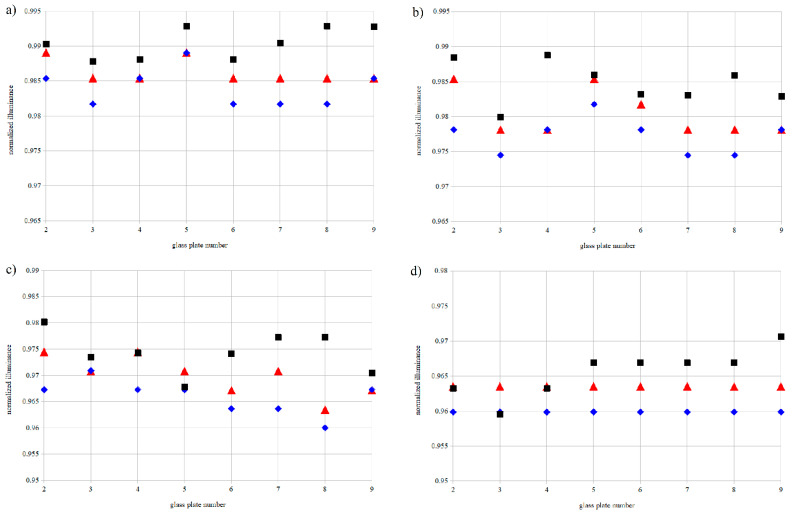
Normalized illuminance for glass plates polluted with soil 1 (■), soil 2 (♦), and soil 3 (▼) at different surface densities: (**a**) 4.3 g/m^2^, (**b**) 8.5 g/m^2^, (**c**) 12.8 g/m^2^, (**d**) 17.1 g/m^2^.

Results from further analysis carried out for three pollutant types and four different surface densities of dust are presented in Figure 11. Normalization was applied with regards to the clean glass plate 1, without hydrophobic coating, which corresponds to value 1. Such an approach enabled coating type analysis of transmittance losses for pollutant-covered glass. For every amount of soil there was a slight drop in light intensity passing through slides, and lower amount of soil did seem to have a positive effect in regaining some of the transparency. Smaller values of dust densities provided some level of difference in illuminance between various pollutant types; however, it never exceeded 1%.

## 6. Conclusions

Decreasing the efficiency of photovoltaic modules caused by the deposition of pollutants on their surface is a significant problem, causing the reduction of generated energy, and thus the decrease of economic viability. An alternative approach to manual cleaning is modification of the cover material and implementing self-cleaning films on glass. It would be useful for application in PV installations on both a large scale and a small scale.

A general conclusion can be drawn from the presented literature review that glass transparency is not impacted, irrespective of if such a coating is hydrophilic or hydrophobic. This, however, is observed for research carried out at an especially small scale, as it was performed under laboratory conditions. Some negative impact on the transparency value was noted when taking into consideration coatings provided by local companies. It is recommended to ask manufacturers for providing details of their product—namely, if they performed glass transmittance analysis before and after thin film application. If such evaluation is available, prosumers are able to choose in full awareness the product with the best possible properties.

## Figures and Tables

**Table 1:** Glass plate numbering and corresponding reference plate without coating (1) as well as plates with hydrophobic coatings (2–9), with information about the manufacturer.

No.	Product	Description of the Coating	Manufacturer
1	No coating	-	-
2	Nano Window	Suitable for: glass Expected durability: 1 year Price of 100 mL: 7.37 USD	Hadwao Nanotechnologia
3	Nano Window Plus	Suitable for: glass Expected durability: 3 years Price of 100 mL: 11.39 USD	Hadwao Nanotechnologia
4	Nano Solar	Suitable for: glass coverage in PV modules and solar collectors Expected durability: 1 years Price of 100 mL: 9.25 USD	Hadwao Nanotechnologia
5	Nanocape Solar	Suitable for: glass coverage in PV modules and solar collectors Expected durability: not specified Price of 100 mL: 9.38 USD	Nanosolution
6	Eco hydrophobic impregnate	Suitable for: glass coverage in PV modules and solar collectors; ceramic roof tiles Expected durability: 1–3 years Price of 100 mL: 27.88 USD	H2O Nanotechnology
7	Nanostone Home Panel PV protection	Suitable for: glass coverage in PV modules and solar collectors Expected durability: 2 years Price of 100 mL: 31.90 USD	P&K J. Marciniak P. Bzukała
8	Nanostone Home Windows protection	Suitable for: glass Expected durability: 3 years Price of 100 mL: 29.97 USD	P&K J. Marciniak P. Bzukała
9	Nanostone Home Shower protection	Suitable for: glass; ceramic Expected durability: 2 years Price of 100 mL: 26.54 USD	P&K J. Marciniak P. Bzukała

**Table 2:** Numbering of soil samples collected from photovoltaic modules, laboratory hall, and three various locations in Gdansk.

Soil Number	Description	Coordinates
1	Taken from roads nearby the Chemistry C GUT building	54°37″ N 18°62″ E
2	Taken from a sandy beach area near the walking pier in Gdansk	54°41″ N 18°64″ E
3	Taken from a sandy beach area near the tram loop in Gdansk	54°41″ N 18°62″ E

## Data Availability

The data presented in this study are available on request from the corresponding author.
